# Evaluating the Role of *PD1* and *MTNR1B* Gene Variants in Breast Cancer Susceptibility: A Case–Control Study in Bangladesh

**DOI:** 10.1002/jcla.70086

**Published:** 2025-07-30

**Authors:** Farhan Jamil, Mohammad Mahfuz Enam Elahi, Al‐Shahriar Evan, Sakif Ahamed Khan, Israt Jahan Annee, Furhatun‐Noor, Ankita Islam, Rana Tabassum, Md. Aminul Haque

**Affiliations:** ^1^ Research Lab Rufaida BioMeds Dhaka Bangladesh; ^2^ American International University‐Bangladesh Dhaka Bangladesh; ^3^ School of Pharmacy BRAC University Dhaka Bangladesh

**Keywords:** breast cancer, cancer risk, *MTNR1B* gene, oncology, *PD1* gene, SNP

## Abstract

**Objectives:**

Breast cancer is one of the most common malignancies in women. The *PD1* and *MTNR1B* gene polymorphisms have been extensively studied for their potential role in cancer susceptibility. This study aimed to investigate associations between breast cancer risk and *PD1* (rs36084323) and *MTNR1B* (rs10830963) polymorphisms.

**Methods:**

A case–control study was conducted with 112 breast cancer patients from Dhaka Cancer and General Hospital, Bangladesh, and 124 age‐ and sex‐matched healthy controls. Genotyping of *PD1* (rs36084323) and *MTNR1B* (rs10830963) polymorphisms was performed using the Polymerase Chain Reaction–Restriction Fragment Length Polymorphism (PCR‐RFLP) method.

**Results:**

The genotype distributions for both SNPs adhered to Hardy–Weinberg equilibrium (HWE). The *PD1* GG genotype was more prevalent in controls, indicating a protective effect, whereas the GG genotype of *MTNR1B* showed no statistically significant association with breast cancer risk. The recessive model of *PD1* (GG vs. AG + AA) demonstrated a lower odds ratio (0.2873), while the recessive model of *MTNR1B* (2.307) suggested a potential risk. Dominant models for both genes (AG + GG vs. AA for *PD1* and CG + GG vs. CC for *MTNR1B*) showed statistically significant associations with breast cancer susceptibility.

**Conclusion:**

The *PD1* GG genotype exhibited a significant protective effect against breast cancer, while the *MTNR1B* CG genotype was associated with reduced risk, but GG showed no correlation. Larger studies across diverse populations are recommended to validate these findings.

## Graphical abstract

The investigation revealed that the PD1 GG genotype exhibited a significant protective effect, substantially reducing the likelihood of breast cancer development. The genotype, CG of the *MTNR1B* gene, was connected with a lessened likelihood of breast cancer development, whereas the GG genotype showed no statistically significant correlation.

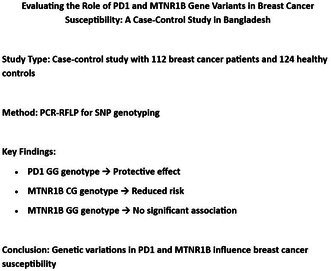

## 1 Introduction

Cancer remains a major global health issue despite advances in diagnosis and treatment. It is the second leading cause of death worldwide, with metastatic cases accounting for most fatalities [1, 2]. Breast cancer, the most common malignancy in women, accounts for over 10% of annual cancer diagnoses and is a leading cause of female cancer deaths [3]. Reducing the incidence of breast cancer relies heavily on addressing the well‐established risk factors [4]. In 2022, 2.3 million women were diagnosed with breast cancer, and 670,000 died from the disease. While its incidence increases with age, breast cancer affects women worldwide [5]. Disparities exist based on human development levels—1 in 12 women in high‐HDI (Human Development Index) nations will develop breast cancer, with a 1 in 7 mortality rate, compared to 1 in 27 and 1 in 48 in low‐HDI countries. Bangladesh has one of the highest breast cancer rates in Asia, with 19.3 cases per 100,000 women aged 15–44, the most common cancer in this group [6].

A large portion of the breast cancer heritability is attributed to single‐nucleotide polymorphisms (SNPs) [7]. Changes in gene expression and function caused by genetic polymorphisms can alter the functions of genes and, in rare cases, lead to disease vulnerability [8]. Therefore, polymorphisms in the genes that regulate immune responses affect the proper functioning of T lymphocyte activation and proliferation, leading to the development of disease [9]. Activated T cells produce the immunoregulatory molecule programmed cell death protein 1 (*PD1*), which regulates the negative aspects of T cell activation and maintains peripheral tolerance during immunological responses [10]. Several tumors have been shown to have immune system avoidance mechanisms that include upregulation of *PD1* [11, 12, 13]. The *PD1* (*rs36084323*) is a single nucleotide polymorphism (A>G) in the promoter region of the PDCD1 gene, which encodes for the *PD1* immune checkpoint protein and has been studied to investigate its association with cancer risk and immune regulation [10]. A meta‐analysis including 10 studies with over 4400 cancer cases and 5100 controls has shown that the (*rs36084323*) A>G polymorphism is linked to an overall decrease in cancer risk in Asian populations [10]. Additionally, a 2011 case–control study in Chinese Han women noted that the GG genotype of *rs36084323* was less frequent in breast cancer patients, indicating a protective effect [14]. However, the results were not universally conclusive and varied across ethnicities; therefore, extensive research is required for further investigation [14]. Moreover, previous studies have suggested that blocking the *PD1* pathway can lead to increased antitumor T‐cell response, and various immunotherapy clinical trials using antibody‐mediated *PD1* blockade are underway in patients with various cancers [15].

The melatonin receptor 1B (*MTNR1B*, rs10830963) gene contains a frequent G risk allele that has been linked to changes in melatonin production and signaling, leading to impaired anticancer effects of melatonin [16]. It was initially postulated and later confirmed that the rs10830963 genotype determines breast and prostate cancer risks, as melatonin has anti‐cancerogenic characteristics [16]. There is mounting evidence linking circadian misalignment, which may occur as a consequence of factors such as social jet lag, night shift work, or lack of sleep, to the development of cancer, particularly hormone‐dependent malignancies like breast and prostate cancer [17, 18, 19, 20, 21]. The principal endocrine signal that transmits the brain's circadian rhythm information to the cells and tissues in the periphery is melatonin, which is a N‐acetyl‐5‐methyltryptamine [18, 19, 20]. Typically, changes in sleep habits are linked to dysregulation of circulating melatonin levels, since melatonin's principal role is to regulate the sleep–wake cycle and seasonality [21]. Numerous other physiological functions may be regulated by melatonin, including endocrine control, neuroprotection, antiaging, inflammation, and immunological modulation. Melatonin's potential effects on cancer's onset, progression, and therapy have therefore been more widely acknowledged [16, 22]. While genome‐wide association studies (GWAS) have investigated the association between the rs10830963 G allele and the susceptibility of polycystic ovarian syndrome and type 2 diabetes, studies on the association between rs10830963 and cancer are limited [23, 24].

Genetic susceptibility to diseases like breast cancer can vary significantly across ethnic groups due to differences in allele frequencies, gene–environment interactions, and lifestyle factors. Therefore, it is essential to evaluate specific single nucleotide polymorphisms (SNPs) within diverse populations to understand their relevance in distinct genetic backgrounds. To address this gap, our study aimed to examine the association of two polymorphisms—*PD1* (rs36084323) and *MTNR1B* (rs10830963)—with breast cancer risk in the Bangladeshi population, where genetic data on breast cancer susceptibility remain limited. To the best of our knowledge, this is the first study investigating these SNPs in relation to breast cancer in this population. We believe our findings provide valuable population‐specific insights and contribute to the broader understanding of genetic risk factors for breast cancer across different ethnic groups.

## 2 Materials and Methods

### 2.1 Materials

TaKaRa Taq DNA polymerase mastermix (PMM), restriction enzymes, and DNA ladders were supplied by TAKARA BIO INC. (Japan). SeaKem LE Agarose was procured from Lonza (USA), and Tris‐borate‐EDTA (TBE) buffer was sourced from Solarbio Life Sciences (China). The DNA extraction kit was provided by Favorgen (USA), while Midori Green was purchased from Nippon Genetics (Europe). Primers were obtained from Macrogen (Korea), and restriction enzymes were sourced from New England Biolabs (USA).

### 2.2 Study Subjects

All research subjects were verified to be devoid of any substantial physical or neurological disorders after thorough physical, neurological, and laboratory assessments. Healthy controls (HCs) and breast cancer (BC) patients were recruited from Dhaka, Bangladesh. A total of 112 patients with breast cancer and 124 age‐ and sex‐matched healthy controls were recruited between April 1, 2023, and June 30, 2023. Breast cancer patients were recruited from the Dhaka Cancer and General Hospital, situated on Sat Masjid Road, Dhaka. We carefully selected breast cancer patients and healthy controls from the same population to reduce selection bias. Controls were matched as closely as possible to cases in terms of age and demographic background. Laboratory personnel performing genotyping were blinded to the case/control status of the samples to avoid observer or measurement bias. The research used meticulously organized inpatient and outpatient facilities for participant assessment. An accredited oncologist evaluated breast cancer patients according to recognized diagnostic criteria, while concurrently assessing the healthy controls. Genotyping analysis was performed at the Rufaida BioMeds facility in Dhaka, Bangladesh. To maintain participant anonymity, the authors were denied access to personally identifying information during and subsequent to data collection.

### 2.3 DNA Extraction and Genotyping

Blood specimens from all subjects were obtained in tubes containing EDTA. Genomic DNA was collected for the single nucleotide polymorphism (SNP) analysis using a commercial genomic DNA extraction kit (Favorgen, USA) in accordance with the manufacturer's instructions. The obtained blood samples were lysed, and the lysate was meticulously placed into binding column tubes. The DNA was then washed with several buffers and eluted with nuclease‐free water. The isolated DNA was preserved at −20°C.

The quality of the isolated DNA was assessed by 1% agarose gel electrophoresis. A thermal cycler (miniPCR, USA) was applied to conduct the polymerase chain reaction‐restriction fragment length polymorphism (PCR‐RFLP) experiment for the detection of SNPs at designated genomic loci. Table 1 delineates the thermal cycling parameters and primer specifications used to amplify the target areas. The PCR reaction comprised 10 μL of PCR 2× master mix, 2 μL of DNA at 100 ng/μL, 2 μL of forward primer at 5 pmol/μL, 2 μL of reverse primer at 5 pmol/μL, and 4 μL of ddH2O. Amplification success was verified through 1% agarose gel electrophoresis using TaKaRa Taq DNA polymerase mastermix.

**TABLE 1:** Primer sequences, thermal conditions and amplified products' size.

Gene, SNP	Primers' sequence	Thermal condition	Fragments, bp
*PD1* rs36084323	F 5′‐CTCACATTCTATTATAGCCAGGACCTCC‐3′ R 5′‐TAAGATAAGAAATGACCAAGCCCAC‐3′	5 min of denaturation at 98°C, then 35 cycles of 98°C for 30s, 55°C for 30s, and 72°C for 40s. The final cycle had a 5 min extension at 72°C	290
*MTNR1B* rs10830963	F 5′‐ATGCTAAGAATTCACACCAGCT‐3′ R 5′‐CACAGTGCAGACTGTTTTCTAATC‐3′	5 min of denaturation at 98°C, then 40 cycles of 98°C for 40s, 52°C for 40s, and 72°C for 30s. The final cycle had a 5 min extension at 72°C	125

Abbreviations: F, Forward; R, Reverse.

The PCR products were subjected to digestion with MspI and BsrI restriction enzymes at 60°C for 1 h each, comprising 8 μL of PCR amplified product, 1 μL of 10X QuickCut buffer, and 1 μL of enzyme. The enzymes were used to examine SNPs at rs4245739 and rs1380576, respectively. The digested products were seen on 3% agarose gels stained with Midori Green. To guarantee the dependability of the findings, duplicate testing was performed on 25% of the whole sample population.

### 2.4 Ethical Consideration

The research received approval from the Institutional Review Board (IRB) of BRAC University (Approval No. BRACUIRB120220005).

Informed written permission was secured from all participants, who were guaranteed the ability to withdraw from the research at any moment without repercussions. All operations were executed in compliance with the criteria and restrictions specified in the sanctioned study protocol.

### 2.5 Statistical Analysis

Statistical analysis was performed using R software (version 4.4.1; R Foundation for Statistical Computing, Vienna, Austria; https://www.r‐project.org/). Hardy–Weinberg equilibrium was tested using the ‘HardyWeinberg’ package. Pearson's Chi‐squared tests were used to check the association between categorical variables across different genotype groups. Odds ratios (ORs) with 95% confidence intervals (CIs) were calculated using multivariate logistic regression models, incorporating age and BMI as covariates.

## 3 Results

### 3.1 Association of *PD1* (rs36084323) Polymorphism With Breast Cancer

The genotype and allele distributions of *PD1* (rs36084323) polymorphism among breast cancer cases (*n* = 112) and controls (*n* = 124) were analyzed, and their association was assessed through different genetic models (Table 2). The Hardy–Weinberg equilibrium (HWE) test revealed that both cases and controls followed equilibrium (*p* > 0.05).

**TABLE 2:** Association between breast cancer and PD1 (rs36084323) polymorphism (Multivariate Logistic Regression adjusted for age and BMI).

Allele	Cases (*n* = 112)	HWE		Controls (*n* = 124)	HWE		Genetic models	Adjusted OR	95% CI	*p*
		*X* ^2^	*p*		*X* ^2^	*p*				
AA Homozygote (197 and 93 bp)	55.35%	0.182	0.669	38.71%	0.478	0.488	Additive model 1 (AG vs. AA)	1.12	0.56–2.21	0.745
							Additive model 2 (GG vs. AA)	0.31	0.54–1.16	< 0.001
AG Heterozygote (290, 197, and 93 bp)	25%			15.32%			Dominant model (AG + GG vs. AA)	0.50	0.29–0.85	0.011
GG Homozygote (290 bp)	19.64%			45.97%			Recessive model (GG vs. AG + AA)	0.29	0.15–0.52	< 0.001
							Over dominant model (AG vs. GG + AA)	0.97	0.53–1.77	0.915

*Note:* *p >* 0.05 obeyed Hardy–Weinberg equilibrium.

The homozygous AA genotype (197 and 93 bp) was more prevalent in breast cancer cases (55.35%) compared to controls (38.71%). The AG heterozygous genotype (290, 197, and 93 bp) was present in 25% of cases and 15.32% of controls. Interestingly, the homozygous GG genotype (290 bp) was significantly higher in controls (45.97%) compared to cases (19.64%).

**FIGURE 1:**
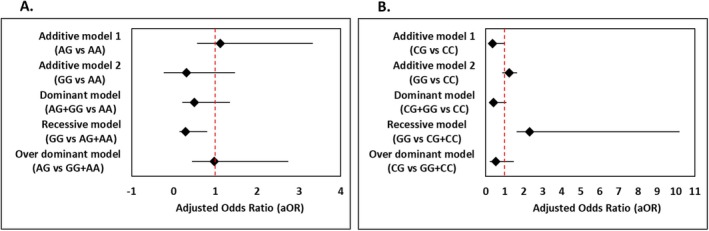
Forest plot. Association between breast cancer with (A) *PD1* (rs36084323) polymorphism and (B) *MTNR1B* (rs10830963) polymorphism.

In the additive model 1 (AG vs. AA), the odds ratio (OR) was 1.1409 (95% CI: 2.283–0.570), indicating no significant association (*p* = 0.709) (Figure 1A). However, additive model 2 (GG vs. AA) showed a significant protective association with an OR of 0.2988 (95% CI: 0.555–1.160, *p* < 0.001), suggesting that the GG genotype may have a protective effect against breast cancer (Figure 1A).

The dominant model (AG + GG vs. AA) also demonstrated a significant association with an OR of 0.5093 (95% CI: 0.855–0.303, *p* = 0.01), further supporting the protective role of the GG genotype (Figure 1A). Similarly, the recessive model (GG vs. AG + AA) revealed an OR of 0.2873 (95% CI: 0.515–0.160, *p* < 0.001), reinforcing the protective association of the GG genotype (Figure 1A). The over‐dominant model (AG vs. GG + AA) yielded an OR of 0.9649 (95% CI: 1.847–0.504, *p* = 0.914), indicating no significant association (Figure 1A).

### 3.2 Association of *MTNR1B* (rs10830963) Polymorphism With Breast Cancer

The genotype and allele frequencies of *MTNR1B* (rs10830963) polymorphism were also assessed among breast cancer cases and controls (Table 3). The HWE analysis showed that both groups adhered to equilibrium (*p* > 0.05).

**TABLE 3:** Association between breast cancer and MTNR1B (rs10830963) polymorphism (Multivariate Logistic Regression adjusted for age and BMI).

Allele	Cases (*n* = 112)	HWE		Controls (*n* = 124)	HWE		Genetic models	Adjusted OR	95% CI	*p*
		*X* ^2^	*p*		*X* ^2^	*p*				
CC Common homozygote (125 bp)	46.42%	0.009	0.921	25.8%	0.245	0.620	Additive model 1 (CG vs. CC)	0.37	0.21–0.65	< 0.001
							Additive model 2 (GG vs. CC)	1.23	0.35–0.42	0.750
CG Heterozygote (125, 105, and 20 bp)	46.42%			70.96%			Dominant model (CG + GG vs. CC)	0.41	0.25–0.69	0.001
GG Rare homozygote (105 and 20 bp)	7.14%			3.22%			Recessive model (GG vs. CG + CC)	2.31	0.68–7.89	0.182
							Over dominant model (CG vs. GG + CC)	0.54	0.32–0.93	0.025

*Note:* *p >* 0.05 obeyed Hardy–Weinberg equilibrium.

The CC homozygous genotype (125 bp) was found in 46.42% of cases and 25.8% of controls. The heterozygous CG genotype (125, 105, and 20 bp) was present in 46.42% of cases and 70.96% of controls. The homozygous GG genotype (105 and 20 bp) was identified in 7.14% of cases and 3.22% of controls.

In the additive model 1 (CG vs. CC), a significant protective association was observed with an OR of 0.363 (95% CI: 0.635–0.208, *p* < 0.001) (Figure 1B). However, additive model 2 (GG vs. CC) did not show a significant association (OR = 1.230, 95% CI: 0.419–0.342, *p* = 0.750) (Figure 1B).

The dominant model (CG + GG vs. CC) exhibited an OR of 0.401 (95% CI: 0.693–0.232, *p* = 0.001), indicating a significant protective role of the CG + GG genotypes (Figure 1B). The recessive model (GG vs. CG + CC) had an OR of 2.307 (95% CI: 7.884–0.675, *p* = 0.182), showing no significant association (Figure 1B). The over‐dominant model (CG vs. GG + CC) showed a significant association with an OR of 0.541 (95% CI: 0.926–0.316, *p* = 0.025), indicating a protective effect (Figure 1B).

### 3.3 Comparative Analysis of *PD1* (rs36084323) and *MTNR1B* (rs10830963) Polymorphisms

A comparative analysis of the two polymorphisms under study highlights the differential role of *PD1* and *MTNR1B* genetic variants in breast cancer susceptibility. The *PD1* GG genotype demonstrated a strong protective effect, significantly reducing the risk of breast cancer development. In contrast, for the *MTNR1B* gene, the CG genotype was associated with a decreased likelihood of developing breast cancer, while the GG genotype did not show a statistically significant correlation.

Notably, the recessive model of *PD1* (GG vs. AG + AA) exhibited a lower OR (0.2873) compared to the corresponding recessive model of *MTNR1B* (2.307), suggesting that *PD1* GG may play a more crucial role in breast cancer prevention. The dominant models for both genes (AG + GG vs. AA for *PD1* and CG + GG vs. CC for *MTNR1B*) exhibited statistically significant associations, further indicating a potential genetic influence on breast cancer risk.

These findings suggest that *PD1* and *MTNR1B* polymorphisms may contribute differently to breast cancer susceptibility. While *PD1* GG appears to have a protective function, the role of *MTNR1B* polymorphisms requires further investigation to understand its precise impact on cancer risk. Future research should explore these genetic variations in larger, ethnically diverse cohorts to validate these associations and clarify the underlying biological mechanisms.

### 3.4 Socio‐Demographic History

The demographic characteristics of breast cancer patients (*n* = 112) and healthy controls (*n* = 124) used in our research are presented in Table 4. The mean ages of cases were 46.9 ± 12.5 years, and the mean ages of controls were 47.8 ± 13.1 years, with no statistically significant difference observed between them (*p* = 0.58). Additionally, the mean BMI was comparable between the two groups (25.7 ± 4.9 vs. 25.2 ± 5.1 kg/m^2^; *p* = 0.32). Moreover, in terms of occupation, the distribution of housewives, working women, and students did not differ significantly between the cases and control groups (*p* = 0.46). Furthermore, the average number of children was also quite similar between the two groups (2.13 ± 0.91 for cases vs. 2.09 ± 0.87 for controls; *p* = 0.45).

**TABLE 4:** Demographic characteristics of breast cancer cases and healthy control.

Variable	Cases (*n* = 112)	Controls (*n* = 124)	*p* *
Age (years)	46.9 ± 12.5	47.8 ± 13.1	0.58
BMI (kg/m^2^)	25.7 ± 4.9	25.2 ± 5.1	0.32
Occupation			
Housewife	38 (33.9%)	44 (35.5%)	0.46
Working women	68 (60.7%)	73 (58.9%)	
Student	6 (5.4%)	7 (5.6%)	
Number of children	2.13 ± 0.91	2.09 ± 0.87	0.45

*Note:* Data are shown as mean ± SD (median) for continuous variables and % (*n*) for categorical variables.

* *p*‐values: *t*‐test for continuous variables (age, BMI, number of children); Chi‐squared test for occupation.

## 4 Discussion

This study examines the impact of *PD1* (rs36084323) and *MTNR1B* (rs10830963) polymorphisms on breast cancer risk in 112 breast cancer patients and 124 healthy controls in the Bangladeshi population. The study found that the GG genotype of the *PD1* polymorphism is associated with a protective effect, whereas specific genotypes of the *MTNR1B* gene, particularly the CG heterozygous genotype, may exert a protective effect against breast cancer.

Breast cancer remains the leading cause of cancer‐related deaths among women globally [25]. Early detection and genetic insights are crucial for mitigating its impact. *PD1* (rs36084323) is a promising candidate for breast cancer susceptibility research, as variations in this gene may alter expression and function, influencing cancer risk [26]. The *PD1* GG genotype significantly reduces breast cancer risk, with the recessive model (GG vs. AG + AA) showing a lower odds ratio (0.2873) [27]. The main models for (AG + GG vs. AA for *PD1*) showed statistically significant associations, indicating a genetic influence on breast cancer risk. According to a meta‐analysis conducted in the Asian population, the *PD1* (rs36084323) polymorphism was shown to decrease cancer risk, particularly for epithelial ovarian cancer and could serve as a potential biomarker to enhance the screening of high‐risk cancer patients [10]. The study reported that *rs36084323*'s G allele is linked to reduced cancer risk in the Asian population with an OR of 0.79 and urged for the screening of the AA genotype for early detection [10]. Conversely, a 2022 pilot study investigating *PD1* polymorphism in breast cancer patients found no association with *PD1* (*rs36084323*) and particular breast cancer subtypes that included luminal A, luminal B, HER2 positive, and triple negative breast cancer [28]. As the *PD1* (*rs36084323*) is an intronic mutation in the promoter region, it might affect the binding affinity of transcription factors, thus disrupting the activation of the gene and influencing the progression of malignant diseases such as cancer [29]. Conversely, Jiao et al. [30] reported that SLE patients with the GG genotype exhibited increased *PD1* mRNA expression, indicating that this SNP may vary by disease. *PD1* is an immune checkpoint receptor that inhibits T cell activation and enhances tumor evasion when it is activated; therefore, reduced *PD1* expression provides enhanced antitumor immunity. However, the results regarding the *PD1* (rs36084323) polymorphism on breast cancer susceptibility are not universal across all ethnicities, as this polymorphism has been found in the Asian population with the highest incidence; therefore, further investigation across all ethnicities is required for conclusive results.

Conversely, various potential pathogenic mechanisms may elucidate the connection among *MTNR1B* rs10830963 and breast cancer. In our study, the CC genotype was observed in breast cancer cases (46.42%) at a higher frequency compared to controls (25.8%), whereas the CG genotype was more commonly observed in controls (70.96) than in cases (46.24) suggesting a potential protective effect. According to a large UK Biobank cohort study with over 216,000 participants, the *MTNR1B* rs10830963 polymorphism was not significantly associated with breast cancer [16]. However, women carrying the G allele with moderate evening chronotype and late chronotype exhibited increased susceptibility to breast cancer [16]. This suggests that females with the G allele and later chronotypes are at higher risk of developing breast cancer, probably due to the circadian rhythm disruption that decreases melatonin's anticancer functions [16]. Additionally, the Shanghai Breast Cancer Study with over 2000 cases and controls also found no significant association between rs10830963 and breast cancer susceptibility [31]. From a biological viewpoint, the G risk allele in rs10830963 is corresponded with reduced insulin production and increased fasting glucose levels, which may directly promote breast cancer growth through hyperglycemia [24]. In breast cancer, the G allele interacts with late chronotypes and increases risk by disrupting melatonin's suppression of estrogen receptor signaling [16]. Furthermore, the G allele may influence MT2 expression in breast tissues, leading to changes in the central circadian clock and peripheral oscillators, including the appearance of the clock genes period 1 (Per1) and period 2 (Per2), which are acknowledged as tumor suppressor genes [24, 32, 33]. However, in our study, the GG genotype was relatively rare in both groups, so further investigation with a larger sample size is required for a conclusive result.

The demographic data of our research showed no significant differences between breast cancer patients and healthy controls in terms of age, BMI, occupation, or number of children. These findings suggested that the case and control group were well matched, reducing the chance of confounding due to these factors. This lack of significant differences in BMI and the susceptibility of breast cancer aligned with the findings from previous studies that were done to assess the effect of BMI on breast cancer risk in the Bangladesh population [34]. However, other studies have reported that higher BMI is a risk factor for breast cancer, especially in postmenopausal women; therefore, further research is needed for assessment [35]. Furthermore, the similarity in the number of children between the case and control suggests that reproductive history does not have any significant effect on breast cancer risk.

Our study has some important limitations that should be addressed. Firstly, our sample size was relatively small, which may hinder the statistical power of our findings. Secondly, we did not consider other important risk factors, for example, lifestyle and environmental factors that participants may have been exposed to, despite their significant impact on breast cancer susceptibility. Therefore, large‐scale and well‐designed studies with different ethnicities are required to confirm our findings.

## 5 Conclusion

The present study provides compelling evidence that *PD1* (rs36084323) and *MTNR1B* (rs10830963) polymorphisms may influence breast cancer susceptibility. The *PD1* GG genotype appears to have a protective effect, significantly reducing the risk of breast cancer, while the *MTNR1B* CG genotype also demonstrates a potential protective role. However, further studies with larger, diverse populations are warranted to confirm these findings and to explore the functional implications of these genetic variations in breast cancer development.

## Author Contributions

Conceptualization: Md.A.H.; data curation: F.‐N. and A.I.; formal analysis: S.A.K. and A.‐S.E.; investigation: F.J., I.J.A. and M.M.E.E.; methodology: Md.A.H.; supervision: Md.A.H.; writing – original draft: M.M.E.E. and Md.A.H.; writing – review and editing: R.T.and Md.A.H.

## Ethics Statement

The study was approved by the Institutional Review Board (IRB) of BRAC University (Approval No. BRACUIRB120220005).

## Conflicts of Interest

The authors declare no conflicts of interest.

## Data Availability

All relevant data are within the manuscript.
